# The Healing Environment for Healthcare Staff in Paediatric Settings: A Cross-National Semi-Structured Survey on Healthcare Staff Satisfaction

**DOI:** 10.3390/ijerph22091444

**Published:** 2025-09-17

**Authors:** Silvia Mangili, Beatrice Pattaro, Andrea Brambilla, Gaia Ferraguzzi, Cristiana Caira, Stefano Capolongo

**Affiliations:** 1 Department of Architecture, Built Environment and Construction Engineering (DABC), Design & Health Lab, Politecnico di Milano, 20133 Milan, Italy; silvia.mangili@polimi.it (S.M.); beatrice.pattaro@polimi.it (B.P.);; 2 Department of Biomedical Science for Health, University of Milan, 20122 Milan, Italy; 3 Department of Architecture and Civil Engineering, Chalmers University of Technology, 412 96 Göteborg, Sweden

**Keywords:** paediatric hospitals, healthcare settings, survey, staff satisfaction

## Abstract

Background: The existing literature emphasises that the hospital environment plays a critical role in the experiences of patients and healthcare workers. To better understand the perspectives of healthcare staff in such settings, a questionnaire-based study was conducted at two paediatric hospitals: Vittore Buzzi Hospital (Milan, Italy) and Queen Silvia Children’s Hospital (Gothenburg, Sweden). Objective: The study had two main objectives: to collect feedback from healthcare staff via a post-occupancy evaluation focused on perceived environmental quality and to compare staff perceptions across different geographical and cultural contexts. Methods: A semi-structured survey tool consisting of 42 closed-ended items (40 of which used a 5-point Likert scale) and 2 open-ended questions was employed. The items assessed the presence and quality of specific environmental and functional features. Results: The findings reveal significant contrasts between the two hospitals. Staff at the Swedish hospital reported higher satisfaction levels across most areas, whereas the Italian hospital demonstrated significant shortcomings. Common concerns in both hospitals included limited space for medical and administrative staff and the absence of private offices for doctors. Conclusions: This study confirms that environmental quality affects not only patients, but also staff, impacting their satisfaction, perceived efficiency, and emotional well-being. The contrasting outcomes highlight the significant influence that differences in architectural design, spatial layout, and investment in staff-oriented spaces can have on the day-to-day experiences of healthcare professionals.

## 1. Introduction

The physical environment of healthcare settings plays a pivotal role not only in determining patient outcomes but also in supporting the well-being, performance, and satisfaction of healthcare professionals [1]. This influence is particularly pronounced in paediatric hospitals, where the emotional and operational demands on staff are uniquely high [2]. Paediatric healthcare workers must address the complex medical needs of young patients while simultaneously providing psychological support to families, often within environments not originally designed to meet the holistic needs of staff and patients alike [1,2,3,4].

Environmental stressors such as excessive noise, overcrowded workspaces, inadequate lighting, and limited access to restorative areas contribute significantly to staff fatigue, psychological stress, and burnout [1,2,5]. Conversely, thoughtfully designed physical environments—characterized by optimal spatial layout, natural daylight exposure, acoustic comfort, and dedicated staff respite areas—have been linked to enhanced teamwork, higher job satisfaction, and improved quality of care delivery [6,7].

Recent research has shifted from a predominantly patient-centred perspective to a more integrative approach encompassing healthcare workers’ experiences, particularly through post-occupancy evaluations (POEs) and evidence-based design (EBD) methodologies [4,8,9]. This paradigm shift reflects a growing recognition that staff-centred design interventions improve both workplace conditions and clinical outcomes. Diverse methods—including observational studies, focus groups, and survey-based tools—have been developed to capture healthcare workers’ feedback on environmental quality.

Among these, semi-structured surveys have emerged as a versatile instrument capable of collecting both quantitative and qualitative data regarding perceived environmental characteristics. These tools facilitate standardization while allowing for open-ended responses that inform actionable design improvements [4]. Nonetheless, research specifically addressing staff satisfaction with paediatric hospital environments remains limited and fragmented. Few studies comprehensively include the perspectives of diverse healthcare roles or examine differences across cultural and architectural contexts, limiting the generalizability of findings [10,11].

Moreover, existing assessment tools tend to focus on immediate satisfaction metrics without integrating forward-looking design insights, which are critical for guiding the development of next-generation paediatric healthcare environments. To bridge these gaps, the present study designs and implements a semi-structured survey exploring staff satisfaction with the built environment in two culturally and architecturally distinct paediatric hospitals: Vittore Buzzi Hospital in Milan, Italy, and Queen Silvia Children’s Hospital in Gothenburg, Sweden.

### Healthcare Workers in Children’s Hospitals

The NEXT (Nurses Early Exit) study highlighted the causes of resignations among healthcare workers. These include working hours, a poor quality of life, intense pressure due to demanding work schedules and responsibilities, and constant harassment, bullying, violence and discrimination [12]. Other causes include a lack of rest areas and green spaces. Stress factors for staff therefore derive from various areas, including factors arising from work tasks such as pressure due to fast-paced work rhythms and deadlines, information overload, contradictory work instructions, and constant interruptions and other forms of disturbance from colleagues, patients, visitors, or family members; factors arising from the professional role such as excessive responsibility or lack of gratification; and environmental factors such as noise, electric shocks, heat, cold, draughts, lack of staff space and rest rooms, long distances between functional areas, and the presence of toxic substances and biological agents [13,14,15].

Working with sick children can be extremely difficult emotionally. Maintaining emotional detachment can be a challenging task, and witnessing a child’s suffering can cause significant emotional stress. In this context, healthcare professionals may experience higher occupational stress than those in other work settings. 

Occupational stress, known as burnout [16], is the result of the interaction between individuals and their working conditions. It manifests as mental and physical exhaustion caused by repeated critical issues in the workplace. This state is not only a personal reaction to suffering experienced by patients, but also a consequence of dissatisfaction with working conditions.

Hospitals can contribute to this malaise through poor organization and adverse environmental conditions. These include the poor availability and maintenance of equipment, as well as uncomfortable environmental conditions such as cramped spaces, inadequate lighting, excessive noise, and physical and social isolation, not to mention interpersonal conflicts. 

These factors make the job more physically and mentally demanding. If the working environment is unfavourable, healthcare workers may experience work overload, affecting their ability to provide quality care to patients, as well as their personal well-being [17].

The workload in a hospital can be divided into two main categories: physical and mental. 

Perceived limited control can result in physical stress, and this can be influenced by the visual control conditions allowed by the spatial layout. Situations involving limited visual control of patients or a lack of control over ward entrances can be significant sources of stress. Other situations that can generate physical stress include heavy lifting and awkward postures, which are often caused by the inadequate ergonomic design of furniture and equipment. The spatial characteristics of the ward, such as the distances travelled daily, can influence the physical demands placed on staff. Spaces that are too small or poorly designed can hinder activity flow and increase physical stress.

Mental fatigue is closely related to stress. Excessive fragmentation of activities caused by spatial disorganization that does not align with staff’s operational sequences can overload cognitive abilities and cause mental stress [3,4,11,14,17]. 

In summary, the spatial and organizational characteristics of the ward can influence both physical and mental stress in the hospital environment, highlighting the importance of a carefully designed layout to improve the well-being and performance of healthcare personnel.

Designating spaces solely for healthcare personnel, where they can engage in training, research activities, clinical case studies, and discussions with colleagues, as well as providing a dedicated lunch break area equipped with a kitchen, tearoom, and a space for reading and recreation, can alleviate the physical and psychological demands of their work.

## 2. Materials and Methods

### 2.1. Survey Design

The survey is a semi-structured instrument comprising a Likert-scale questionnaire and two open-ended questions. The seven sections (Sample Data, Rooms, Inpatient Area, Operating rooms, Intensive Care Unit, Common Areas, and Administrative Areas) composing the survey includes 42 closed-ended items, of which 40 are based on a 5-point scale designed to assess the presence or absence of specific environmental and functional features. The final item of the Likert section addresses the respondent’s overall job satisfaction, while the concluding open-ended section invites qualitative feedback—both positive and critical—regarding the physical work environment.

At the beginning of each interview, the scale and its interpretation were clearly explained to ensure full comprehension. An introductory letter accompanied the survey, emphasizing the anonymity of responses and outlining the scope of the study.

### 2.2. Administration

The survey was administered face to face, using a paper-based format to ensure focus and engagement, and the data were analysed in an excel file. It was conducted in Italian at Vittore Buzzi Hospital and in English at Queen Silvia Children’s Hospital. To maintain consistency and ensure comparability of results, all interviews were carried out by the authors of this study.

### 2.3. Objectives

The survey served three main purposes:

1. Post-occupancy evaluation tool, leveraging perceived quality to gather feedback from healthcare staff;
2. Comparative analysis of staff perceptions between two paediatric hospitals with different geographical and cultural contexts;
3. Source of design recommendations for a checklist aimed at guiding the development of the ‘Paediatric Hospital of the Future.’

### 2.4. Sample

The survey was conducted face-to-face with a total of 60 participants: 30 from Vittore Buzzi Hospital and 30 from Queen Silvia Children’s Hospital. The sample included healthcare personnel only—physicians, nurses, healthcare assistants, staff supporting paediatric play, educators, and administrative staff—excluding patients and family members. Most responses were from nursing and administrative staff rather than physicians in both locations. Participant ages ranged from 34 to 62 years, with 43 women (72%) and 17 men (28%) in the sample.

The study employed an observational approach, where an initial narrative review of case studies informed the development of a field survey. This survey was conducted at Vittore Buzzi Hospital in Milan during January and February 2024 and at Queen Silvia Children’s Hospital in Gothenburg in April 2024.

### 2.5. Structure of the Survey

The survey was semi-structured, consisting of a comprehensive Likert-scale questionnaire with 42 items. The scale included five response options ranging from ‘Very Satisfied’, indicating that the structural characteristic was present as expected, to ‘Very Dissatisfied’, indicating that the structural criterion was not met. The intermediate options captured partial satisfaction, neutrality, unfamiliarity and partial dissatisfaction.

Thirty-nine of the total items focused on specific structural characteristics across different hospital areas, including patient rooms, inpatient areas, operating rooms, intensive care units, common areas, and administrative spaces. These sections examined criteria such as privacy, colour schemes, furnishings, paediatric rehabilitation and play facilities, parent comfort, staff areas, orientation strategies, flexibility, patient safety, continuity between indoor and outdoor spaces, green views, environmental sustainability, natural lighting, sound insulation, space dimensions, infection control, communication between healthcare staff and parents, visiting hours, and technology in the operating theatre.

The remaining three questions assessed general satisfaction levels, with one addressing overall satisfaction and two taking the form of open-ended questions to capture qualitative feedback.

The measurement scale was explained at the start of each interview to ensure clarity. Participants received a letter emphasizing the anonymity of responses and the research’s purpose. Conducted in person, the survey was administered in Italian at Vittore Buzzi Hospital and translated into English at Queen Silvia Children’s Hospital, maintaining engagement and accuracy in responses.

## 3. Results

**Figure 1:**
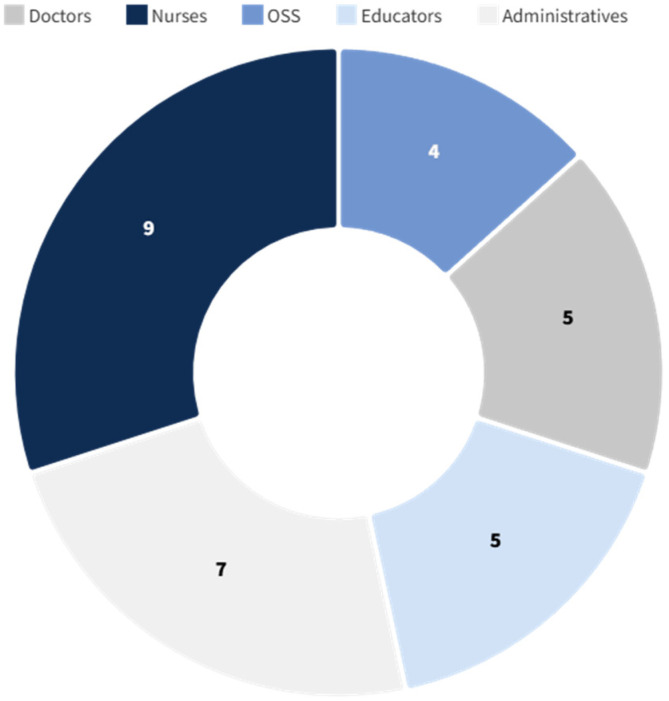
Survey respondents—Vittore Buzzi Hospital (Milan, Italy).

**Figure 2:**
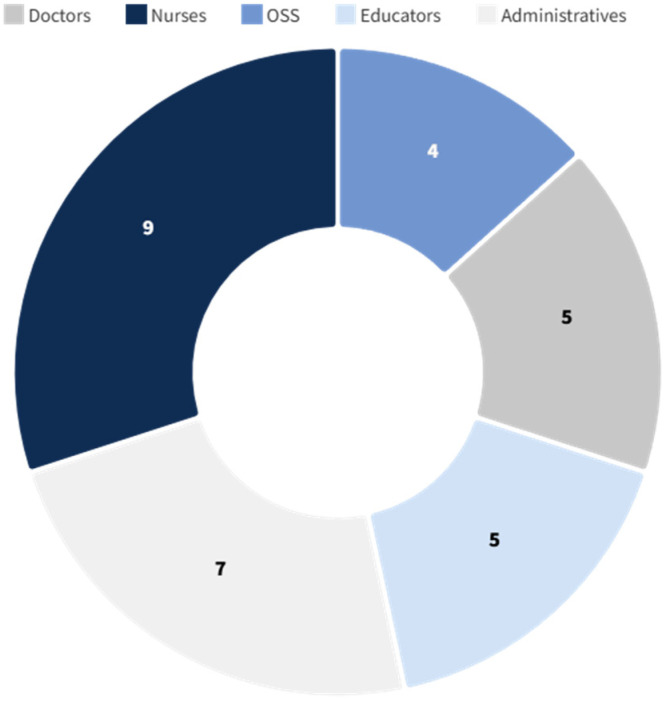
Survey respondents—Queen Silvia Children’s Hospital (Gothenburg, Sweden).

The survey was successfully submitted to a sample of 60 healthcare professionals, equally distributed between Vittore Buzzi Hospital (Milan, Italy) and the Queen Silvia Children’s Hospital (Gothenburg, Sweden). The sample included a broad spectrum of staff roles—physicians, nurses, nursing assistants (OSS), play and education specialists, and administrative personnel—but excluded patients and caregivers (Figure 1 and Figure 2), focusing exclusively on internal perceptions of the built environment. 

All 60 individuals invited to participate accepted, resulting in a 100% response rate. The majority of respondents in both hospitals were nurses and administrative staff, with a smaller number of medical doctors. The age of participants ranged from 34 to 62 years, with a gender distribution of 43 women (72%) and 17 men (28%).

### Data Analysis

The response revealed significant differences in responses between the two hospitals, particularly in areas related to inpatient rooms, common areas, and intensive care units. 

In analysing the data collected from the questionnaire responses, the mode was selected as the most appropriate measure of central tendency. This choice is justified by the nature of the data, which consists of categorical responses rather than numerical values. Specifically, the analysis aims to highlight the most frequently selected answer among participants at each hospital.

The responses marked with an ‘X’ represent 26 identical answers out of 30 participants at Queen Silvia Children’s Hospital (87%), while the ‘x’s represent the remaining 13%. Those marked with an ‘O’ correspond to 22 similar answers out of 30 participants at Vittore Buzzi Hospital (73%), while the ‘o’s represent the remaining 27%. These figures indicate a clear predominance of particular responses within each group (Table 1, Table 2, Table 3, Table 4, Table 5 and Table 6).

The purpose of the following tables is to underscore the most common sentiment or experience reported; the mode effectively captures and communicates this trend. Unlike the mean or median, which are more suitable for quantitative data, the mode provides a meaningful summary in contexts where the frequency of specific categories is of primary interest.

## 4. Discussion

Significant differences in response were observed across several criteria, including natural lighting and acoustic insulation in patient rooms, availability and quality of family accommodation, spatial flexibility and privacy, access to nature and green spaces, dedicated zones for play, education and rehabilitation, and facilities for medical staff, such as break areas, offices, and training spaces.

The data shows that Queen Silvia Children’s Hospital scored consistently higher across most of these categories, particularly in terms of environmental comfort and family-centred design. These findings are consistent with an expanding body of interdisciplinary research that emphasises the important role of environmental conditions in the healing process. Elements such as spatial design, lighting, ventilation, and access to nature are increasingly recognised for their impact on patient mood, stress reduction, and recovery speed, as well as their aesthetic value [1,2,6,7]. In modern paediatric care, such considerations are vital as they affect the well-being of children, the emotional resilience of families, and the effectiveness of care teams. 

However, as highlighted in the introduction, the physical environment is not only a patient issue but also a crucial factor in supporting the well-being, performance, and satisfaction of healthcare professionals [1,2]. Environmental stressors such as noise, inadequate lighting, overcrowded workspaces, and the absence of restorative areas are well documented contributors to staff fatigue, stress, and burnout [1,2,5]. Conversely, thoughtfully designed features—such as ergonomic layouts, acoustic comfort, daylight exposure, and dedicated staff areas—have been linked to improved teamwork, job satisfaction, and quality of care [6,7]. Despite these strengths, negative feedback was received by both hospitals regarding the limited space allocated to medical and administrative personnel, as well as the lack of private offices for doctors. These criticisms reveal a bigger problem in how modern hospitals are designed. Often, hospitals put medical functions first and do not think enough about the mental and emotional needs of staff and patients. The result is a clinical environment that can feel cold or impersonal, which may hinder the space’s therapeutic potential. The NEXT study, for example, highlighted the role of poor environmental conditions—including inadequate rest areas and lack of access to green spaces—in driving staff dissatisfaction and even professional resignation.

This study therefore adds to existing evidence by confirming that organisational and spatial characteristics of hospital wards directly influence both physical and mental stress for healthcare workers [3,4,11,14,17]. Factors such as fragmented workflows caused by poorly designed layouts, long distances between functional areas, and limited opportunities for privacy exacerbate both physical strain and cognitive overload. Conversely, the provision of dedicated staff spaces for rest, reflection, and professional exchange could play a decisive role in reducing occupational stress and preventing burnout [16].

In this regard, the cross-cultural comparison between the two hospitals underscores how architectural strategies and investment choices impact staff experiences differently. While the Swedish context demonstrates the benefits of embedding staff-oriented design principles, both cases reveal the persistent under-design of staff areas. 

### 4.1. Overall Staff Satisfaction

The final item in the survey asked participants to rate their overall satisfaction with their workplace environment (Table 7). At Vittore Buzzi Hospital, responses were split:

- A total of 18 respondents (60%) stated they were “somewhat satisfied”;
- A total of 12 respondents (40%) reported being “somewhat dissatisfied”;
- No participant selected the extremely positive or negative options.

Conversely, at the Queen Silvia Children’s Hospital, 100% of respondents reported positive satisfaction:

- A total of 27 respondents (93%) were “very satisfied”;
- A total of 3 respondents (7%) were “somewhat satisfied”.

These results confirm the stronger environmental satisfaction at Queen Silvia Children’s Hospital (Table 8), which reflects its more recent infrastructure and design strategies that prioritize both family and staff needs.

### 4.2. Qualitative Feedback

The final section of the survey comprised two open-ended questions, inviting participants to share their positive and negative experiences, as well as any suggestions for future improvements.

Participants at Queen Silvia Children’s Hospital highlighted concerns relating to limited space in medical and administrative areas, the absence of private offices for physicians, and distractions caused by natural light in operating theatres. Some also expressed privacy concerns due to the low-level windows in operating theatres. Another significant issue was the underutilisation of the preoperative room, which was originally designed to allow family members to be present during anaesthesia induction, but which is now rarely used. These points underscore the importance of ensuring that architectural features are well designed and fully integrated into clinical workflows and hospital culture.

At Vittore Buzzi Hospital, frequent criticisms included cramped spaces in clinical and administrative areas, a lack of private offices for doctors, and inadequate facilities, such as locker rooms, for trainees. Respondents also deemed double-bed patient rooms to be inappropriate for long paediatric stays and identified insufficient comfort zones for families and staff within care units. Parking difficulties were identified as an additional logistical concern that caused further stress for staff and visitors alike.

These observations reflect the extent to which environmental features influence clinical processes, staff morale, work–life balance, and perceptions of professional value. The physical layout and atmosphere of a hospital play a crucial role in creating a sense of safety, dignity, and normality for patients, particularly children, and their families. Literature on healthcare design emphasises that environments which promote privacy; comfort; and engagement with nature, art, and play can contribute significantly to recovery and emotional stability.

Furthermore, an absence of adequate support areas for staff, such as private offices, rest zones, and training spaces, can undermine team cohesion and reduce the sense of institutional investment in their well-being. Positive design facilitates healing for patients and strengthens the professional identity and satisfaction of healthcare providers. This is particularly important in paediatric care settings, where interdisciplinary collaboration and emotional sensitivity are essential.

Comparing these two hospitals makes it clear that intentional, human-centred design enhances not just operational efficiency but also the overall caregiving experience. In paediatric contexts, where emotional care is inseparable from medical treatment, thoughtful architecture can support children’s developmental needs, promote resilience, and ultimately improve clinical outcomes. These findings strongly suggest that architectural design should be considered a fundamental element in the planning and delivery of paediatric healthcare services.

## 5. Conclusions

This study sought to address a specific research gap in the literature concerning the impact of paediatric hospital environments on healthcare staff, an area often overshadowed by patient-centred studies. While environmental features are increasingly recognized as contributing to healing processes [18,19], the subjective perceptions of medical personnel—particularly in emotionally and operationally intensive contexts like children’s hospitals—remain underexplored. By conducting a face-to-face survey across two leading European institutions, this research offers new insights into how professionals interpret and evaluate the spaces where they work and care. 

The results confirm that environmental quality is not only a patient issue but also a staff concern, deeply influencing satisfaction, perceived efficiency, and emotional well-being. These findings echo those of Pati et al. and Joseph (2016) [3,20], who emphasized the importance of restorative and supportive design in clinical settings but expand the scope by adopting a comparative, cross-cultural approach and integrating qualitative feedback directly from the field. 

From a practical perspective, the findings can inform architects, hospital planners, and policymakers by identifying concrete spatial priorities—such as improving rest areas, staff-only workspaces, and administrative environments—that directly enhance the daily functioning and well-being of healthcare professionals.

The contrast between the two hospitals—Queen Silvia Children’s Hospital and Vittore Buzzi Hospital—highlights how differences in architectural strategies, operational layout, and investment in staff-oriented spaces can shape the day-to-day experience of healthcare professionals. Despite national and contextual differences, both settings revealed common criticalities related to the under-design of administrative and rest areas, as well as a lack of private workspaces for physicians—confirming concerns raised in prior research [21,22] about the neglect of staff spaces in paediatric hospital design. In conclusion, this research confirms that the quality of space is deeply intertwined with quality of care, not only for patients but also for those who serve them. Listening to the healthcare workforce is an essential step toward creating truly therapeutic environments, where well-being is shared across all actors involved in care.

### 5.1. Limitations

The study displays several inherent limitations. The sample is composed exclusively of staff members, which means that the perspectives of patients and caregivers are excluded. This means that the findings can only be interpreted in terms of professional experience. Additionally, while face-to-face interviews provide rich qualitative data, they may introduce response bias due to social desirability or contextual influences. Furthermore, the two hospitals involved differ significantly in terms of age, structural typology, and healthcare systems, and these differences may affect satisfaction levels independently of design-related factors. Finally, only a selection of the survey questions is presented in this paper; a full statistical analysis of all 42 items is currently underway. 

### 5.2. Future Developments

This study suggests several avenues for future research. Firstly, increasing the sample size to encompass a broader range of hospital types and geographical contexts would enhance the generalisability of the findings. Secondly, involving patients and families, especially in paediatric care, is essential for a comprehensive assessment of the care environment. Another important area of research involves integrating post-occupancy evaluation (POE) frameworks and participatory co-design workshops, as suggested by recent literature on design and health [23], to support more responsive and inclusive planning practices. Additionally, this study can serve as a source of design recommendations for a checklist aimed at guiding the development of the ‘Paediatric Hospital of the Future’, linking empirical insights from staff surveys to practical design guidance. Finally, developing a standardised, validated, and staff-inclusive survey tool that can be adapted to different care settings could provide a much-needed benchmark for evaluating healing environments.

## Figures and Tables

**Table 1:** Rooms.

Rooms	Very Satisfied	Somewhat Satisfied	Neutral	Somewhat Dissatisfied	Very Dissatisfied
Is natural light sufficient to illuminate the room during daylight hours without the need for artificial light?	x	X		O	o
Is proper noise insulation guaranteed?	x	X		O	o
Are there beds for caregivers in the rooms?	X	x		O	o
Room dimension	X	x		O	o
Are there only single rooms?	X	x		o	O
Flexibility of the rooms for emergencies	X	x		O	o
Are there professionals who can help children personalize their rooms during long hospital stays?	X	x		o	O
Do all rooms have double entrance doors?	x	X		O	o
Is there a sterilisation system in all bathrooms?	X	x		o	O
Does every room have a view of natural/green areas?	X	x		o	O

**Table 2:** Inpatient Area.

Inpatient Area	Very Satisfied	Somewhat Satisfied	Neutral	Somewhat Dissatisfied	Very Dissatisfied
Are there several team stations (doctor, nurse, student interns dedicated to a specific group of rooms)?	X	x		o	O
Is there a place outside the hospital room for caregivers to eat?	X	x		O	o
Is there a desk in every ward?	X	x		o	O
Is there access to outdoor space without having to leave the ward?	X	x		O	o

**Table 3:** Operating room.

Operating Room	Very Satisfied	Somewhat Satisfied	Neutral	Somewhat Dissatisfied	Very Dissatisfied
Is there a space dedicated to post-surgery care?	X	x		o	O
Are there waiting areas for parents/caregivers?	X	x		O	o
Is it possible for parents to stay in a dedicated area within the operating theatre during the operation?	X	x		o	O
Is there a pre-operation room where parents can stay with their child during the first stage of anaesthesia?				X and o	O and x

**Table 4:** Intensive Care Unit.

Intensive Care Unit	Very Satisfied	Somewhat Satisfied	Neutral	Somewhat Dissatisfied	Very Dissatisfied
Is it possible for parents/caregivers to access intensive care at any time?	X	x		O	o
Does every operating theatre have its own control room?	x	X		o	O
Flexibility of the room in case of emergency	X	O and x	o		
Are the intensive care rooms single?	X	x		o	O
Is there a bed for parents/caregivers in the ICU room?	X	x		o	O
Is there a waiting room or tearoom for parents?	X	O			
Are there rooms in the ICU department for parents in case of an emergency in the ward so that they can stay in the hospital overnight?	X	x		o	O
Are there isolated rooms in the ICU department with separate entrances in case of infection?	X	x	o	O	
If so, do these rooms have a space with a bed and bathroom dedicated to the parent who is assisting?	X	x		o	O
Does every room have a view of natural/green areas?		X	x	o	O

**Table 5:** Common areas.

Common Areas	Very Satisfied	Somewhat Satisfied	Neutral	Somewhat Dissatisfied	Very Dissatisfied
Is the information desk directly lit?	X	x		o	O
Play area for children	X	x		o	O
Space dedicated to school activities in the event of long hospital stays	X	x	o	O	
Private and shared gym for specific rehabilitation	X	x		o	O
Presence of swimming pool	X	x		o	O

**Table 6:** Administrative area.

Administrative Area	Very Satisfied	Somewhat Satisfied	Neutral	Somewhat Dissatisfied	Very Dissatisfied
Are there any spaces dedicated to medical/nursing training?	X	x		o	O
Are there rest areas equipped with beds and showers available for doctors on night duty?	X	x		O	o
Are there areas available for doctors and nurses to take breaks and relax?	X	x		o	O
Are there private offices for doctors?			x	X and O	o
Is the space in the doctor’s rooms adequate?			x	X and o	O
Was the same importance given to the space for healthcare personnel as to that for patients?		X	x	o	O

**Table 7:** Overall staff satisfaction of Vittore Buzzi Hospital.

Vittore Buzzi	Very Satisfied	Somewhat Satisfied	Neutral	Somewhat Dissatisfied	Very Dissatisfied
Number of Responses	0	18	0	12	0
Percentage	0%	60%	0%	40%	0%

**Table 8:** Overall staff satisfaction of Queen Silvia Children’s Hospital.

Queen Silvia Children Hospital	Very Satisfied	Somewhat Satisfied	Neutral	Somewhat Dissatisfied	Very Dissatisfied
Number of Responses	27	3	0	0	0
Percentage	93%	7%	0%	0%	0%

## Data Availability

The data presented in this study are available on request from the corresponding author due to the large amount of data and privacy concerns involved.
